# Pattern Recognition of Spiking Neural Networks Based on Visual Mechanism and Supervised Synaptic Learning

**DOI:** 10.1155/2020/8851351

**Published:** 2020-10-27

**Authors:** Xiumin Li, Hao Yi, Shengyuan Luo

**Affiliations:** College of Automation, Chongqing University, Chongqing 400044, China

## Abstract

Electrophysiological studies have shown that mammalian primary visual cortex are selective for the orientations of visual stimuli. Inspired by this mechanism, we propose a hierarchical spiking neural network (SNN) for image classification. Grayscale input images are fed through a feed-forward network consisting of orientation-selective neurons, which then projected to a layer of downstream classifier neurons through the spiking-based supervised tempotron learning rule. Based on the orientation-selective mechanism of the visual cortex and tempotron learning rule, the network can effectively classify images of the extensively studied MNIST database of handwritten digits, which achieves 96% classification accuracy based on only 2000 training samples (traditional training set is 60000). Compared with other classification methods, our model not only guarantees the biological plausibility and the accuracy of image classification but also significantly reduces the needed training samples. Considering the fact that the most commonly used deep learning neural networks need big data samples and high power consumption in image recognition, this brain-inspired computational neural network model based on the layer-by-layer hierarchical image processing mechanism of the visual cortex may provide a basis for the wide application of spiking neural networks in the field of intelligent computing.

## 1. Introduction

Pattern recognition is to distinguish different kinds of patterns according to the semantic information of inputs, and it is an important basic problem in the field of computer vision and artificial intelligence. There is a wide range of applications in reality, such as object recognition [1, 2], region detection [3], aurora image analysis [4], and scene categorization [5]. Due to the great research value, researchers have developed many methods for pattern recognition, for example, k-nearest neighbor classifier [6], multilayer perceptron [7], support vector machine [8], and convolutional neural networks (CNNs) [9, 10]. In these methods, CNNs are one of the most commonly used methods which has achieved great success in intelligent computing. Although CNNs implement a biological-inspired network topology, the node models and learning rules are quite different from the real-life biological neural networks. And CNNs often rely on the error backpropagation learning rule, which has been criticized for being biologically unrealistic and requiring massive computing power, time, and energy, and thus make it infeasible for real-time applications, e.g., on mobile devices or autonomous robots [11].

In fact, the learning method based on the error gradient descent lacks biological support. It is known that the human being has an overwhelming advantage in image recognition, which shows that our brain has a unique processing mechanism in visual processing. Therefore, many researchers try to find more biosupported classification methods by imitating the structure and synaptic plasticity learning rules of biological neural networks. Among various temporal learning rules for spiking neural models, several rules have been widely studied, including spike-timing-dependent plasticity (STDP), the tempotron rule, the SpikeProp rule, the SPAN rule, the Chronotron rule, and the ReSuMe rule [12]. Markram proposed the spike-timing-dependent plasticity (STDP) which has been confirmed to explain the brain activities especially for LTP and LTD [13]. STDP learning rule which adjusts the synaptic weights according to the spiking time interval of presynaptic and postsynaptic currents is widely used for unsupervised learning of SNNs [14, 15]. In view of the great success of deep learning, Kheradpisheh proposed a spiking deep convolutional neural network (SDNN) based on STDP rules, which can extract image features with multilayer convolution operation [16], Kulkarni proposed normalized approximate descent to apply error back propagation [17], and they both achieved high performance on the MNIST dataset. In other applications, Seijoon proposed spike Yolo, which is the first time to use the SNN for target detection and has achieved the same performance as CNN [18]. In particular, the tempotron learning is specially designed to solve the binary classification problem of spike trains. Compared with other algorithms, it has stronger pattern recognition ability and can make appropriate decisions based on an error-correcting strategy which adjusts only a few parameters under the effect of supervisory signals. Moreover, different from traditional supervised learning algorithm based on gradient descent, the tempotron learning does not need to explicitly calculate the gradient of the cost function with respect to parameters, which avoids the problem caused by the discontinuity of firings in SNNs.

Besides learning rules, preprocessing of input data and visual attention is also crucial for visual image recognition [19]. In 1959, Hubel and Wiesel inserted a electrode into the cat's visual cortex and projected light and dark pattern on the screen in front of the cat, and they found that some neurons in the visual cortex fired rapidly when the line was at a particular angle, while other angles corresponds to different neurons [20]. This experiment showed that neurons in the visual cortex are selectively responsive to the directions of visual stimulation, and such cells are known as orientation selective cells. Subsequently, Simoncelli established a mathematical model, called the motion energy model [21], for the response of visual cortical areas V1 and MT. And the MT receptive field is sensitive to pattern direction. Recently, Beyeler et al. modify the motion energy model and runs on off-the-shelf graphics processing unit (GPU) successfully [22]. Their simulator CARLsim is written in C/C++ and running on CUDA environment which is an efficient computing platform of NVIDIA, supporting many kinds of spiking neuron models and synaptic plasticity rules [23]. They built a hierarchical feedforward SNN that integrates a low-level memory encoding mechanism with a higher level decision process to perform a visual classification task in real-time. The network was trained by an STDP-like learning rule and finally achieved 92% accuracy in the MNIST database of handwritten digits [24].

In general, although SNNs have strong biological characteristics and have been broadly studied in computational neuroscience, the computing accuracy is lower than that of traditional artificial neural networks. In this article, by applying the motion energy model and orientation-selective mechanism of visual cortex as studied in [24] for the preprocessing of input data, we use the biologically plausible supervised synaptic learning rule named tempotron [25] to replace the STDP-like learning rule to train the output weights. In order to simplify the training process and improve computational efficiency, we propose the single-spike temporal encoding which is one of the simplest temporal encoding for the transform of firing rates to spiking trains before the tempotron learning. Our results show that the this model is very efficient for the MNIST hand-written digit recognition with the recognition accuracy of 96% based on only 2000 training samples. Compared with other image classification methods, our model needs much smaller data samples to achieve high performance of classification.

## 2. Methods

### 2.1. Network Architecture

**Figure 1:**
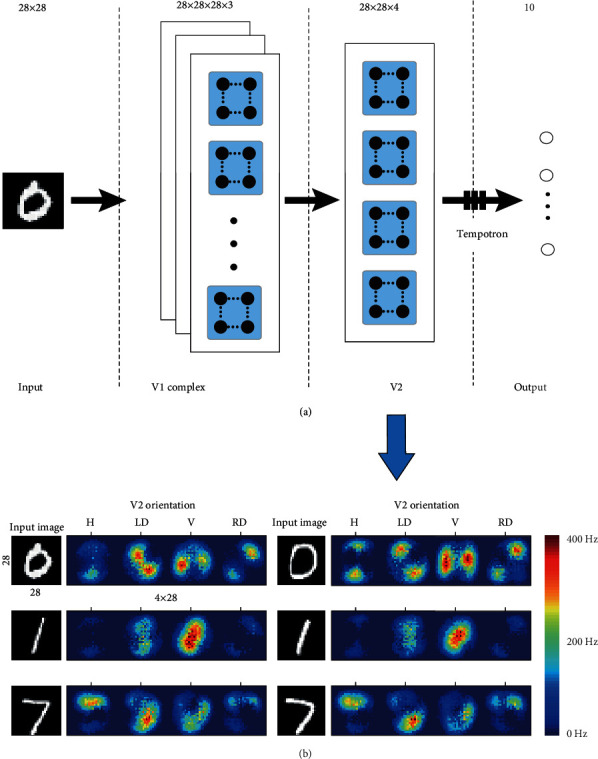
(a) Network architecture. The network is divided into four layers (dotted boxes). The input layer (MNIST grayscale images with 28 × 28 pixels) was fed through a feed-forward network consisting of V1 and V2, which then projected to a downstream population of decision neurons (output layer). The black dot is the Izhikevich model, and the white circle is the leaky integrate-and-fire (LIF) model. The input is the raw pixel value of MINIST grayscale images. V1 simple and complex cell responses were constructed by using the rate-based motion energy model. Simulated spiking neurons in V2 received input only from V1 complex cells and preferentially responded to one of four spatial orientations. There are full connections from the V2 layer to the output layer, with the weights updated by the tempotron learning rule. (b) Some MNIST samples (28 × 28) and their corresponding orientation-selective responses (28 × 112). Each image is spatially expanded into four pool orientation-selective responses of the V2 layer neurons, with each pool of neurons preferentially responded to one of four spatial orientations (horizontal “H,” right diagonal “RD,” vertical “V,” and left diagonal “LD”). Firing rates are color-coded ranging from 0Hz (blue) to 400Hz (red).

The network architecture is shown in Figure 1(a). The network is divided into four layers (dotted boxes). The input layer (MNIST grayscale images with 28 × 28 pixels) was fed through a feed-forward network consisting of V1 and V2, which then projected to a downstream population of decision neurons (output layer). V1 and V2 populations were previously implemented and tested in papers [21–23]. Each image is spatially expanded into four pools orientation-selective responses of the V2 layer neurons, with each pool of neurons preferentially responded to one of four spatial orientations in 45° increments (horizontal “H,” right diagonal “RD,” vertical “V,” and left diagonal “LD” (Figure 1(b)). V1 simple and complex cell responses were constructed by using the rate-based motion energy model. The 28 × 28 pixel images in the MNIST dataset were processed at three different spatial scales resulting in 84 filter responses per pixel. Simulated spiking neurons in V2 received input only from V1 complex cells and preferentially responded to one of four spatial orientations. The orientation layer V2 thus consists of four pools of 28 × 28 neurons (as illustrated in Figure 1(b)). Synaptic weights from V2 to the output layer are trained using the spiking-based tempotron supervised learning rule which will be introduced in the following sections. Figure 1(b) shows some MNIST samples (28 × 28) and their corresponding orientation-selective responses (28 × 112). The orientation layer consisted of four pools of 28 × 28 neurons responding to one of four spatial orientations. Although there are subtle differences in writing for the same digit, the heatmaps are very similar and significantly different from the other digits. For example, digit 1 shows the strongest response to the vertical direction. Firing rates are color-coded ranging from 0 Hz (blue) to 400 Hz (red).

## 3. Motion Energy Model

V1 consists of the V1 simple cell, V1 complex cell, and spiking neurons. V1 cell responses were constructed by using the first stage of the (rate-based) motion energy model [23]. V1 simple cells are modeled as linear space-time-oriented filter whose receptive fields, consisted of 28 space-time orientations (Uniform distribution in sphere), are third derivatives of a Gaussian which are very similar to a Gabor filter. V1 complex cell responses were computed as a weighted sum of simple cell afferents that have the same space-time orientation and phase. Since the motion energy model expected movies rather than static images as an input, we expand the 28 × 28 pixel image into videos (20 frames with each duration of 50 ms) as the input. A visual stimulus was processed at three different spatiotemporal scales (as the three banks depicted in neuron populations in the V1 layer of Figure 1) resulting in 84 filter responses per pixel. Filter responses were then interpreted as the mean firing rates of Poisson spike trains which are the input of V1 spiking neurons.

V2 only consists of spiking neurons which receive the input from V1 spiking neurons and more sensitive to four specified directions in 45° increments (horizontal “H,” right-diagonal “RD,” vertical “V,” and left-diagonal “LD”). The neurons were broadly tuned such that they only response strongest to a specific direction in a Gaussian neighborhood and send an inhibitory projection to the neurons with the antipreferred direction. V2 orientation layer consisted of 3136 neurons correspond to 28 × 28 pixels in four orientations. The orientation information is presented by the firing rate of the V2 neuron population. For complete model description and parameter settings, please refer to [21–23]. Here, V1 and V2 layers are modeled by using the CARLSim SNN simulator platform which is available on the website http://www.socsci.uci.edu/jkrichma/CARLsim/.

## 4. Spiking Neuron Model

The neurons in V1 and V2 are modeled by using the Izhikevich neuron [26], which has both computational efficiency and biological plausibility for simulating large-scale spiking neural networks.

(1) $$\begin{array}{c} \begin{array}{c} dV\left(t\right)/dt=0.04V{\left(t\right)}^{2}+5V\left(t\right)+140-U\left(t\right)+I^{\text{syn}}\left(t\right)\\ dU\left(t\right)/dt=a\left(bV\left(t\right)-U\left(t\right)\right), \end{array}\\ \text{if }V>30\text{mV},\text{ then}\left\{\begin{array}{c} V=c\\ U=U+d \end{array}\right., \end{array}$$

where *V* represents the membrane potential, and *U* is a membrane recovery variable; *I*^syn^ is the external synaptic current through neuron, and the units for the membrane potential *V* and time *t* are mv and ms, respectively. The parameters *a*, *b*, *c*, *d* can be set as different values according to different neuron types. For example, regular spiking (RS) neurons (excitatory neurons) have *a* = 0.02, *b* = 0.2, *c* = −65, *d* = 8, and fast spiking (FS) neurons (inhibitory neurons) have *a* = 0.1, *b* = 0.2, *c* = −65, *d* = 2 [26, 27].

There are two synapse model descriptions: the current-based (CUBA) description uses a single synaptic current term while the conductance-based (COBA) description calculates the synaptic current using more complex conductance equations for each synaptic receptor-type. Both CUBA and COBA current contributions are influenced by the synaptic weight of the synapse [28]. Here, we use the COBA mode. There are four receptor types, that is, AMPA (fast decay), NMDA (slow decay and voltage-dependent), GABA_*a*_ (fast decay), or GABA_*b*_ (slow decay). A spike arriving at a synapse that is postsynaptically connected to an excitatory (inhibitory) neuron increases both *g*_AMPA_ and *g*_NMDA_ (*g*_GABA_*a*__ and *g*_GABA_*b*__). So, *I*_syn_ includes the excitatory current and inhibitory current:

(2) $$\begin{array}{c} \begin{array}{c} I_{\text{syn}}=i_{e}+i_{i}\\ i_{e}=i_{\text{AMPA}}+i_{\text{NMDA}}\\ i_{i}=i_{\text{GAB}{\text{A}}_{a}}+i_{\text{GAB}{\text{A}}_{b}} \end{array}. \end{array}$$

The total current *I*_syn_ for each neuron is given by

(3) $$\begin{array}{c} \begin{array}{ccc} i_{\text{AMPA}}=g_{\text{AMPA}}\left(v-v_{\text{AMPA}}^{\text{rev}}\right) & & \\ i_{\text{NMDA}}=g_{\text{NMDA}}\frac{{\left[v+80/60\right]}^{2}}{1+{\left[v+80/60\right]}^{2}}\left(v-v_{\text{NMDA}}^{\text{rev}}\right) & , & \\ i_{\text{GAB}{\text{A}}_{a}}=g_{\text{GAB}{\text{A}}_{a}}\left(v-v_{\text{GAB}{\text{A}}_{a}}^{\text{rev}}\right) & & \\ i_{\text{GAB}{\text{A}}_{b}}=g_{\text{GAB}{\text{A}}_{b}}\left(v-v_{\text{GAB}{\text{A}}_{b}}^{\text{rev}}\right) & & \end{array} \end{array}$$

where *g* and *v*_rev_ are specific to a particular ion channel or receptor. Synaptic conductances *g* obey the exponential decay and changed when presynaptic spikes arrived.

(4) $$\begin{array}{c} \frac{dg_{r}\left(t\right)}{dt}=-\frac{1}{\tau_{r}}g_{r}\left(t\right)+w\underset{i}{\sum }\;\delta \left(t-t_{i}\right), \end{array}$$

where *δ* is the Dirac delta function, *w* is the synaptic weight, *τ* is the time constant, and *r* is the receptor type (AMPA, NMDA, GABA_*a*_, GABA_*b*_). *t*_*i*_ is the arriving time of presynaptic spikes. In our simulations, we set the parameters as *τ*_AMPA_ = 5ms, *τ*_NMDA_ = 150ms, *τ*_GABA_*a*__ = 6ms, and *τ*_GABA_*b*__ = 150ms.

## 5. Single-Spike Temporal Encoding for V2

**Figure 2:**
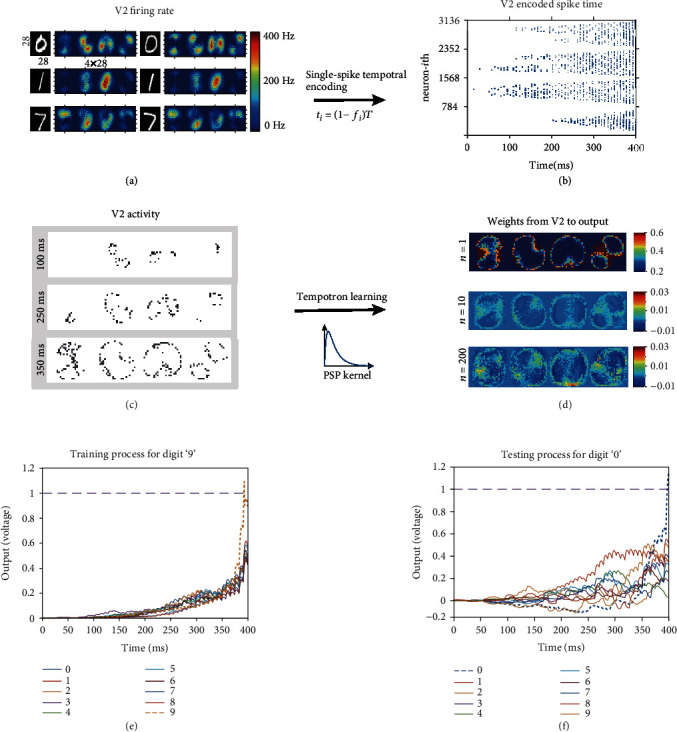
(a) Firing rates of four pool orientation-selective V2 neurons for different digit images. (b) Spike trains for V2 neurons after *t* = 100,250,350ms. (d) Synaptic weights from the V2 layer to the output layer after tempotron learning with *n* = 1,10,200 training samples for the digit “0.^”^ Note that firing activities of the edge neurons of each pool of the orientation population in V2 as shown in (c) (bottom panel) are quite similar with the weight matrix after learning. (e, f) The voltage accumulations of output neurons during the training and testing process, respectively.

Since the orientation information in the V2 layer is presented by firing rates of the neuronal population (shown in Figure 1(b)), it cannot be directly transmitted to the output neurons for the spiking-based supervised learning. Here, we adopt the latency coding [29] for the transform of V2 firing rates to spiking trains. Latency coding is one of the simplest temporal encoding. It encodes information of the response time in the encoding window, which is related to external stimulus. According to the characteristics of neurons, i.e., the larger the input, the earlier the neuron discharges, and no firing without stimulation, the input information is encoded into a delay time relative to the initial time and mapped to a predefined time window. In order to simplify the training process and improve computational efficiency, we normalize the firing frequencies of V2 neurons and encode them into a spiking train in the time window between 0 and *T* with each neuron fires only once (i.e., single-spike temporal encoding). V2 neurons with high firing rates would result in earlier encoded spiking time, whereas neurons with low firing rates would firing later or no firing at all (as shown in Figures 2(a)–2(c)). In this way, the spike train of the encoded V2 layer is sparse and much efficient for tempotron learning. The spiking time of the *i*th V2 neuron is obtained by

(5) $$\begin{array}{c} t_{i}=\left(1-f_{i}\right)T. \end{array}$$

Here, *f*_*i*_ is the normalized firing frequency of the *i*th (*i* = 1, 2, 3136) neuron in the V2 layer. The length of time window is *T* = 400ms. After encoding, the higher the firing frequency, the earlier the spike time *t*_*i*_. For example, if the *i*th V2 neuron has no firings, its encoded spike time *t*_*i*_ is equal to *T*. Note that the spiking time *T* is not included in the calculations of synaptic learning.

## 6. Tempotron Supervised Learning

Following the previous implementation of V1 and V2 layers and the single-spike temporal encoding, we train the synaptic weights from V2 to the output layer for the recognition of the MNIST database using a spiking-based supervised learning rule, called the tempotron learning proposed in [25]. The V2 orientation layer is fully connected to the output layer which is plastic in Figure 1. The output layer consists of 10 leaky integrate-and-fire (LIF) neurons, corresponding to 10 types of digits (0, 1, ⋯, 9). There are 3136 × 10 connections (presynaptic 3136 neurons times postsynaptic 10 output neurons), and the synaptic weights *w* are a matrix of 3136 × 10. The subthreshold membrane voltage of the decision/output layer is a weighted sum of the postsynaptic potentials (PSPs) from all incoming spikes of the V2 layer [25]:

(6) $$\begin{array}{c} V_{j}\left(t\right)=\overset{N}{\underset{i=1}{\sum }}\;w_{ij}\underset{t_{i}}{\sum }\;K\left(t-t_{i}\right)+V_{\text{rest}}, \end{array}$$

where *t*_*i*_ is the spiking time of the *i*th neuron (*i* = 1, 2, ⋯, *N*, *N* is the number of presynaptic V2 neurons), and *w*_*ij*_ is the synaptic weights from the *i*th V2 layer neuron to the *j*th output neuron. *K*(*t* − *t*_*i*_) is the normalized PSP contributed by each incoming spike:

(7) $$\begin{array}{c} K\left(t-t_{i}\right)=V_{0}\left(\frac{\exp\left(-\left(t-t_{i}\right)\right)}{\tau }-\frac{\exp\left(-\left(t-t_{i}\right)\right)}{\tau_{s}}\right),t_{i}<t, \end{array}$$

Here, *τ* and *τ*_*s*_ denote decay time constants of membrane integration and synaptic currents, respectively. The factor *V*_0_ normalizes PSP kernels to 1. Input spike arrives in the time *t* which locates in the time window [0, *T*]. When *V*(*t*) crossed the firing threshold (here, *V*_threshold_ = 1), the neuron elicits a spike, then *V*(*t*) exponentially decays by shunting all incoming spikes that arrived after the output spike (for details, please see [25]). Here, we take *V*_rest_ = 0, *V*_0_ = 2.12, *τ* = 16ms, and *τ*_*s*_ = 4ms.

The update of weights follows the below rules:

(8) $$\begin{array}{c} \Delta w_{j}=\left\{\begin{array}{cc} \lambda_{+}\underset{t_{i}<t_{\max}}{\sum }\;K\left(t_{\max}-t_{i}\right) & \text{if }P_{+}\text{error},\\ -\lambda_{-}\underset{t_{i}<t_{\max}}{\sum }\;K\left(t_{\max}-t_{i}\right) & \text{if }P_{-}\text{error},\\ 0 & \text{otherwise}. \end{array}\right. \end{array}$$

Here, *t*_max_ is the time when the membrane potential *V*(*t*) of the decision layer reached the maximal value. The constant *λ*_+_ and *λ*_−_ denotes the updating value of synaptic weights for each input spike. Δ*w*_*j*_ is the change value of synaptic weight projecting to the *j*th output neuron, *j* = 1, 2, ⋯, 10. The classical tempotron rule is used in the binary classification problem. Each input pattern belongs to one of two classes (labeled *P*_+_ and *P*_−_). Assume that when input belongs to the *P*_+_ (*P*_−_) pattern, the output neuron should spike (not spike). But if the input belongs to *P*_+_ and the output neuron does not spike, then *P*_+_ is the error, so is the same for *P*_−_. The tempotron learning is to modify the synaptic weights whenever an error occurs.

Here, we explore the tempotron learning for multiclassification task of the 10-digit recognition. During the training process, the correct output neuron which belongs to the current input pattern is labeled as *P*_+_ and *P*_−_ for the other nine outputs. Therefore, the voltage *V* of the labeled neuron should pass the firing threshold for spiking, while the voltages *V* of the other nine neurons should be under the firing threshold (not spike). If this condition is not met, then the *P*_+_ error and *P*_−_ error occurred. The weights will be updated until the outputs meet the condition. The average number of iterations for learning is usually around 5 for *P*_+_ and 1 for *P*_−_. The initial values of weights from the V2 layer to the decision layer are quite small (0.001), thus weights are gradually increased to trigger the correct output neuron firing.

## 7. Results

In order to evaluate the computational performance of our model, we applied it to the extensively studied MNIST database of handwritten digits. The simulation comprised three stages: a preprocessing stage in which the MNIST dataset was converted into orientation responses, a training phase, and a testing phase. The preprocessing stage was performed only once initially. Each image is spatially expanded into four pool orientation-selective responses of the V2 layer neurons, with each pool of neurons preferentially responded to one of four spatial orientations in 45° increments (horizontal “H,” right diagonal “RD,” vertical “V,” and left diagonal “LD” (Figure 2(a)). After the single-spike temporal encoding, firing rates in the V2 layer are transformed into spike trains with each neuron that has only one spiking time as shown in Figure 2(b). Since the firing rate of each V2 neuron is encoded as a single spike with the spiking time proportional to the firing rate, the encoded spike trains of the four pools of orientation-selective neurons look like four triangles, where the image center of the four orientation population corresponding to the highest intensity of the V2 response shows the earliest encoded spiking time. Figure 2(c) shows the spiking neurons at three different times after the single-spiking temporal encoding, and we can see that center neurons with the highest firing rates fire at the earliest times, while edge neurons with low firing rates fire near the end of time window.

During the training stage, the change of synaptic weights Δ*w* is calculated according to the kernel equation (8), where updating changes exponentially declined with the increase of time interval between the spiking time and the time when the membrane potential of the output layer reached the maximal value, i.e., *t*_max_. Since the number of presynaptic V2 spikings gradually increase during each time window, the output neurons reach the maximal value at the end of each time window. Therefore, as shown in Figure 2(d), synaptic updates from center neurons to output neurons can be ignored due to the large time interval between their spiking times *t*_*i*_ and *t*_max_. Only weights from edge neurons to output neurons are significantly increased. By comparing Figure 2(c) and Figure 2(d), we can clearly see that firing activities of the edge neurons of each pool of the orientation population in V2 are quite similar with the weight matrix after learning. In this way, after training the temporal information of the V2 activity as shown in Figure 2(c), it is successfully transferred and saved as spatial information in the synaptic weights from V2 to the outputs as shown in Figure 2(d). For each sample image, synaptic weights are updated only once at the end of each time window. With the increase of training samples *n* = 1,10,200, more weights from edge neurons to the outputs are enhanced, making the weight image become more fuzzy and polarized.

Figures 2(e) and 2(f) illustrate a sample run for which the accumulation of voltage caused the labeled output neuron to cross the threshold. Since the encoded information of the presynaptic V2 firings are concentrated in the later part of time window as shown in Figure 2(b), the voltages rise from zero gradually during the whole time window. During the training process, the voltage of the labeled output neuron with input belongs to *P*_+_ is accumulated repeatedly due to the increase of weights until it crosses the threshold. While the weights targeting to the other output neurons are almost not updated, keeping the other voltages under threshold. After training all the weights are fixed, the output neuron having the highest membrane voltage is taken as the final recognition result. From this figure, we can notice that for both training and testing process, the voltage of the correct output is significantly integrated and increased to reach to the threshold during the last 50ms of the time window. This means that only presynaptic spikes from the edge neurons which firing at the end of time window can be successfully propagated to the output layer. And only the correct output neuron connected by the previously learned weights from the corresponding four pools of V2 neurons receives the most information and shows the most rapid accumulation to reach to the threshold.

**Figure 3:**
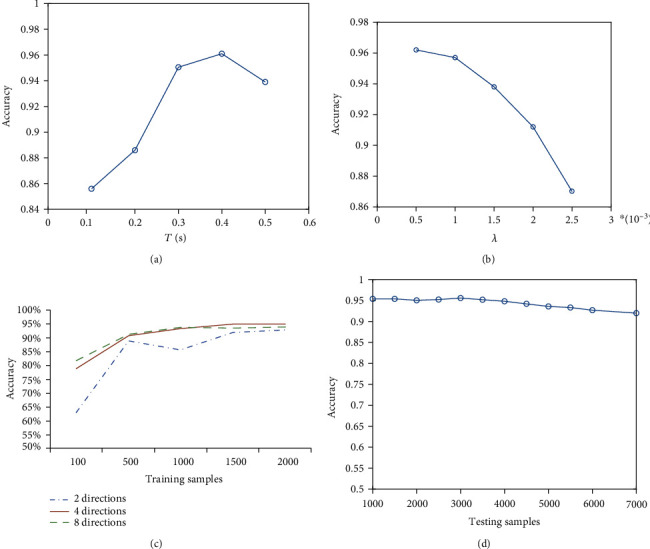
The influence of the time window *T* (a) and weights updating amount *λ* (b) on the classification accuracy of our model. In this paper, we choose the optimal value as *T* = 400ms and *λ* = 0.001. (c) The influence of training samples on the computational performance of this model with 2, 4, or 8 directions in the V2 layer, where testing samples are 1000. (d) The influence of testing samples on the computational performance, where training samples are 2000.

We also examine the influence of the parameter time window *T* and weights updating amount *λ* as shown in Figures 3(a) and 3(b). Due to the nonlinear dynamical characteristics of synaptic integration, if the time window *T* is too small, presynaptic information from V2 neurons cannot be completely propagated to the output layer, but if it is too large, since the synaptic current decays exponentially, it will lose much useful information after a long time of attenuation. Therefore, the value of *T* should be moderate to ensure that less information is lost in the process of synaptic transmission; otherwise, the performance of the network will be seriously reduced. In this paper, *T* is set to be the optimal value 400ms. For the parameter of weights updating amount *λ*, the updating time of synaptic weights will increase and prolong the training time when *λ* is too small, while if *λ* is too large, the synaptic weights will change greatly in each adjustment step, which will reduce the learning ability of the network and lead to the decline of recognition accuracy. Therefore, considering both of the recognition accuracy and time consumption, *λ* is set to be 0.001 in this paper.

Figure 3(c) demonstrates that the larger the number of orientations/directions in the V2 layer is, the smaller training samples are needed for achieving high recognition accuracy. For the cases of 4 directions and 8 directions, the accuracy can reach to 93% based on only 500 training samples. Besides, the computational performance of this model is very robust with the increase of testing samples as shown in Figure 3(d), with the training sample of 2000 for the 4 direction cases.

In Table 1, we compare our model with some other spiking-based neural networks on the task performance of the MNIST classification. Compared with the 60000 training samples and 10000 testing samples needed in the other methods, our model needs only 2000 training samples and 1000 testing samples and almost has no much loss of accuracy. In this paper, we used the tempotron learning rule to replace the STDP-like learning rule in [24], which further improved the classification accuracy on the MNIST dataset. The results show that the method adopted in this paper can achieve high classification performance with much smaller training samples.

**Figure 4:**
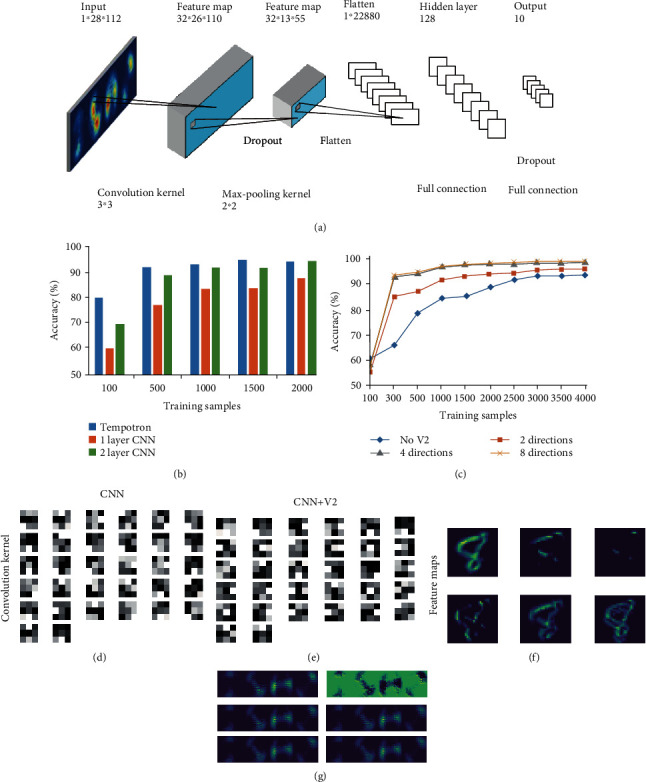
(a)The architecture of the convolutional neural network (CNN). The convolutional layer has 32 convolution kernels of size 3 × 3. The pooling layer has kernels of size 2 × 2. The output layer is a fully connected layer of 128 × 10, which is mapping to 10 classes. (b) The comparison of classification accuracy between our model and CNN with one or two convolutional layers in the tasks for different number of training samples. The testing sample is 1000. (c) The performance of 1 layer CNN by adding V2 layer with two, four, or eight directions before the convolutional layer. The two directions are horizontal and vertical while the four and eight directions are in 45° or 22.5° increments, respectively. The convolutional kernels (d, e) and feature maps (f, g) for CNN (1 convolutional layer) with or without the V2 layer are compared.

Since convolutional neural networks (CNN) are most commonly used for image recognition in recent years, we specifically compared the computational accuracy of our model and traditional CNN with one or two convolutional layers in the task with different training sample numbers. The CNN architecture is shown in Figure 4(a).  Figure 4(b) demonstrates that the accuracy increases with the increase of training samples for both our model and CNNs, but the performance of our model is always better than CNNs especially for small training samples. Noted that when training set is 500, the classification accuracy of our model achieves a high performance of 93% while the accuracy of CNN with two layers is only about 86%. Although CNN can increase the accuracy by adding convolution layers, it still cannot achieve good performance for small samples.

In order to examine whether the preprocessing of the V2 layer can improve the performance of CNN, we add the V2 layer with two, four, or eight directions before the convolutional layer of CNN (Figure 4(c)). The two directions are horizontal and vertical while the four and eight directions are in 45° or 22.5° increments, respectively. To avoid too much calculation, only CNN with one convolutional layer is examined by adding the V2 layer. From this figure, we can see that the V2 layer with four or eight directions can more significantly improve the performance of CNN for small training samples than the case of only two directions. When further increasing the number of training samples, the more training sets will lead to larger calculation cost but no significant improvement in the classification accuracy. Figures 4(d)–(g) compare the convolutional kernels and feature maps for CNN (one convolutional layer) with or without the conversion of V2 orientation responses from the image data. We can see that more feature maps for CNN with the V2 layer can be extracted than the traditional CNN, which contributes to the reduction of training samples and improvement of computational performance.

## 8. Conclusion

Here, we present a large-scale model of a hierarchical spiking neural network to perform a visual classification task in real-time. Based on the orientation-selective features of the primary visual cortical neurons and supervised tempotron learning method, we have successfully realized the high performance of MNIST handwriting digit recognition with small data samples. The orientation-selective layer expands the original image into high dimensional information to achieve highly detailed feature extraction, which provides the main contribution to reduce the training samples. Our results also show that the tempotron supervised learning rule is efficient to train the weight matrix between the V2 layer and output layer, which further improves the recognition accuracy. Compared with other image classification methods, our method needs much smaller data samples to achieve high performance of classification. Our results indicate a new brain-inspired computational model based on the visual cortex image processing mechanism to realize high-precision image recognition under small sample data. This work may provide a basis for the wide application of spiking neural networks in the field of intelligent computing, which is valuable and meaningful in both theoretical and applied researches.

## Figures and Tables

**Table 1:** Comparisons with other spiking-based models on the performance of MNIST recognition.

Model	Train	Test	Accuracy (%)
Spiking deep CNN [16]	60000	10000	98.4
Spiking deep NN [30]	60000	10000	98.6
Spiking deep belief network [31]	60000	10000	94.09
SNN+NormAD [17]	50000	10000	98.2
Living NN+STDP [32]	10000	1000	74.7
SNN+STDP [24]	2000	1000	92
V2 layer+tempotron (this model)	2000	1000	96

## Data Availability

The data used to support the findings of this study are included within the article.
